# Prognostic significance of minimal extrathyroidal extension in differentiated thyroid carcinoma: a retrospective cohort study

**DOI:** 10.1016/j.bjorl.2026.101780

**Published:** 2026-03-11

**Authors:** Beatriz G. Cavalheiro, Vergilius José Furtado de Araújo Filho, Leandro Luongo Matos, Ana Kober Leite, Claudio Roberto Cernea, Luiz Paulo Kowalski

**Affiliations:** a Hospital das Clínicas da Faculdade de Medicina da Universidade de São Paulo, Instituto do Câncer do Estado de São Paulo, Head and Neck Surgery Department, São Paulo, SP, Brazil; b Faculdade de Medicina da Universidade de São Paulo, São Paulo, SP, Brazil; c Faculdade Israelita de Ciências da Saúde Albert Einstein e Instituto de Ensino e Pesquisa Albert Einstein, Hospital Albert Einstein, São Paulo, SP, Brazil

**Keywords:** Thyroid neoplasms, Papillary carcinoma, Prognosis, Recurrence, Extracapsular extension

## Abstract

**Objectives:**

To evaluate the impact of minimal Extrathyroidal Extension (mETE) on structural recurrence, prognostic variables, and disease-free survival in patients with Differentiated Thyroid Carcinoma (DTC) treated surgically.

**Methods:**

A retrospective cohort of 629 patients who underwent thyroidectomy for DTC was analyzed. Patients were divided into two groups: Group 1 (n = 413) with tumors confined to the thyroid, and Group 2 (n = 216) with minimal extrathyroidal extension. Clinical, pathological, and biochemical variables were compared between groups. The minimum follow-up period was 30-months or until death, with an average follow-up of 71.5-months. Univariate and multivariate analyses were performed to identify independent predictors of structural recurrence.

**Results:**

Structural recurrence occurred in 6.5% of Group 1 and 22.7% of Group 2 patients (p < 0.001). Group 2 showed significantly higher rates of follicular carcinoma, tumor size > 2 cm, angiolymphatic invasion, pN1 disease, multicentricity, elevated postoperative thyroglobulin (Tg > 10 ng/mL), iodine-refractory disease, distant metastases, and higher late Tg levels (all p < 0.05). Even though the univariate associations were statistically significant, multivariate analysis identified only male sex (p = 0.018), postoperative Tg >10 ng/mL (p < 0.001), and iodine-refractory disease (p < 0.001) as independent predictors of structural recurrence, not mETE. Five-year disease-free survival was 94.1% in Group 1 and 79.2% in Group 2 (p < 0.001).

**Conclusion:**

Minimal extrathyroidal extension was associated with several adverse prognostic factors and worse outcomes in univariate analysis. However, it was not an independent predictor of structural recurrence or reduced disease-free survival in this cohort. These findings highlight the need to understand mETE in the larger context of risk stratification in management of DTC.

**Level of evidence:**

3.

## Highlights

• Minimal ETE is associated with adverse risk features in thyroid carcinoma.
• Patients with minimal ETE had higher recurrence rates in univariate analysis.
• Minimal ETE was not an independent predictor of poor outcomes.
• Risk stratification should not rely solely on the presence of minimal ETE.

## Introduction

Differentiated Thyroid Carcinomas (DTC) are the most prevalent endocrine neoplasms. Its incidence has increased dramatically in recent decades, primarily through Papillary Thyroid Carcinoma (PTC).^1^^,^^2^ Despite the favorable disease-specific survival rates, locoregional and/or distant relapses occur in about 20% of cases, usually within the first decade of follow-up.^3^^,^^4^

The relationship between Extrathyroidal Extension (ETE) and the prognosis is well-established for patients with DTC, especially PTC.^5^ Gross ETE is a variable associated with poor outcomes, and has been correlated with the risk of developing recurrent local disease and distant metastases, as well as an increased risk of dying from the tumor.^6^, ^7^, ^8^

Meanwhile, minimal Extrathyroidal Extension (mETE) ‒ extension into the surgical thyroid capsule or limited to the perithyroidal soft tissue ‒ has been considered less clinically important and its impact on prognosis is debatable. In the current AJCC-UICC TNM (8th edition) staging system, this factor has lost its importance and what was formerly considered pT3 is now considered pT1 or pT2, according to the size of the tumor.^9^ The Initial Risk Stratification System proposed by the American Thyroid Association (ATA) also considers mETE as a not-so-important factor, with an associated risk of structural disease recurrence of 3% to 8%, and a variable that categorizes the patient in the ATA intermediate-risk group for recurrence.^10^ However, some authors pointed to mETE an underestimated risk factor in current clinical practice, including a systematic review and meta-analysis showing that mETE is significantly associated with increased risk of disease recurrence.^11^, ^12^, ^13^, ^14^, ^15^, ^16^

Minimal Extrathyroidal Extension (mETE) is reported in 15%–66% of Differentiated Thyroid Carcinomas (DTC) measuring 1–4 cm.^13^ This variability largely reflects the absence of standardized criteria and the inherent subjectivity of diagnosis, as mETE is not a well-defined histopathologic feature and the thyroid lacks a true anatomic capsule.^17^ This fact futher complicates the discussion.

Therefore, the purpose of the present study was to analyze the impact of minimal extrathyroidal extension as an independent factor on the outcomes of patients undergoing surgical treatment for DTC, as well as to correlate this pathological finding with other potential risk variables.

## Methods

This is an observational and retrospective real world study, part of a research project (#228/14) approved by the Ethics in Research Committee FMUSP. The data were obtained through analysis of the electronic medical records of the patients included in the study.

The eligibility criteria included patients submitted to thyroidectomy for DTC with a minimum follow-up time of 30-months (or until death). The final cohort included 685 consecutive individuals treated between 1998 and 2014. Patients defined with gross invasion into adjacent structures were excluded, and a total of 629 individuals were considered. Thyroid tumors with poorly differentiated or undifferentiated components were not included. Also, only patients who received R0 ressection were considered.

The assessment of mETE was based on the information provided in the original pathology reports. The histological material was not reviewed, as mETE is routinely described and recorded in the institutional pathology reports.

Through clinical, surgical, and histopathological assessments, patients were categorized into two groups: Group 1 (no extrathyroidal extension), comprising tumors confined within the surgical thyroid capsule with no evidence of invasion, and Group 2 (microscopic extrathyroidal extension), comprising tumors with minimal invasion beyond the surgical thyroid capsule, limited to the immediate perithyroidal soft tissues, detectable only on histopathological examination, with no evidence of gross or macroscopic invasion or involvement of strap muscles, in accordance to the American Thyroid Association definition.

Radioactive Iodine (RAI) therapy was administered according to institutional practice at the time of treatment. Since the cohort spans from 1998 to 2014, patients were managed under evolving international guidelines and local protocols, which influenced the indications for RAI administration across different time periods.

The groups were analyzed and compared for their distributions by age and sex, tumor histology, tumor dimensions, presence of regional metastatic disease and distant metastases at diagnosis, multicentricity, angiolymphatic invasion, serum thyroglobulin titles, radioiodine-refractoriness, and outcomes.

Radioiodine-refractory disease was defined according to ATA criteria, including at least one of the following: absence of RAI uptake on post-therapy scans, structural disease progression despite RAI uptake, progression after cumulative activity ≥600 mCi, or lack of response to prior RAI with no expected benefit from further treatment.^10^

Serum basal Thyroglobulin (Tg) and anti-thyroglobulin antibody measurements were standardized as references for biochemical response to the therapy and the final clinical condition. Tg was assessed under thyroid-stimulant hormone suppression (levothyroxine therapy), both in the postoperative period and at one year after initial treatment. Stimulated Tg testing was not systematically performed and therefore was not included in this analysis. TSH suppression was administered to all patients, depending on their risk classification for relapse.^9^ Imaging studies (at least an annual neck ultrasound) were performed, and structural recurrence was determined based on the presence of anatomical neoplastic lesions on physical examination or imaging. Locoregional disease and some distant metastases were submitted to a cytological study to confirm DTC relapse.

Structural Disease-Free Survival (SDFS) was calculated from the date of initial surgery (time origin) until the first documented evidence of structural recurrence or last follow-up. Patients with evidence of persistent structural disease after initial therapy were considered to have an event at time zero for the SDFS analysis.

### Statistical analyses

The values obtained by the study of the quantitative variables were described as mean, median, and Standard Deviation (SD), and the categorical variables were described through absolute or relative (%) frequencies. Comparisons of frequencies between groups were performed by the Pearson Chi-Square test or the Fisher exact test. The Kaplan–Meier method was used in univariate survival analyses and the log-rank test was applied both in the comparison among survival curves and for univariate analyses. The Cox regression model was applied in the multivariate analysis for those variables with p-value <0.20 in univariate analyses, and in estimating the Hazard Ratio (HR) and its 95% Confidence Interval (95% CI). The program Statistical Package for the Social Sciences ‒ SPSS® version 26.0 (IBM®; Endicott, NY, USA) was used in all analyses and the level of statistical significance adopted was below 5% (p-value ≤0.05).

## Results

The demographic, clinical, and pathological characteristics of the cohort are described in Table 1. The mean age at diagnosis was 52-years (SD ± 14.3-years, median of 52-years, ranging from 15 to 88-years), with a predominance of women (87.1%). The tumor diameters ranged from 0.1 cm to 10.5 cm (mean of 1.7 cm, SD ± 1.1 cm, median of 1.2 cm), and only five patients (0.8%) were submitted to partial thyroidectomies. Selective lymph node dissections at the first treatment were performed on 138 patients (21.9%), and 392 (62.3%) received Adjuvant Radiodine Therapy (RAI) after the initial operation. The mean follow-up time was 71.5-months, SD ± 24.9-months, and a median of 69-months. Group 1 consisted of 413 patients (65.7% of the cohort), and, Group 2, of 216 individuals (34.3%).

**Table 1:** Characterization of the patients included in the cohort.

Variable		Series	
		Number of patients	%
Sex	Female	548	87.1
	Male	81	12.9
Age group (year at diagnosis)	<45	191	30.4
	≥45 and <55	159	25.3
	≥55	279	44.3
Histology	Papillary carcinoma	597	94.9
	Follicular carcinoma	29^a^	4.6
	Papillary + Follicular carcinoma	3	0.5
Tumor diameter (cm)	≤2	470	69.9
	>2 and ≤4	108	19.1
	>4	51	10.9
Tumor invasion	Thyroid-limited tumor	413	65.7
	Capsular	34	5.4
	Microscopic extracapsular	182	28.9
Angiolymphatic invasion	Yes	85	13.5
Multicentricity	Yes	281	44.7
Cervical lymph node metastasis (pN1disease)^b^	Yes	109	17.3
Distant metastasis^b^	Yes	17	2.7
Postoperative Tg under TSH suppression^b^	<1 ng/mL	184	37.4
	≥1 ng/mL and <10 ng/mL	224	45.5
	≥10 ng/mL	84	17.1
Basal Tg 1-year after initial treatment^c^	<0.2 ng/mL	469	75.4
	≥0.2 ng/mL and <1 ng/mL	77	12.4
	≥1 ng/mL andz<10 ng/mL	49	7.9
	≥10 ng/mL	27	4.3
Outcomes	Tumor persistence	44	7.0
	Tumor relapse	37	5.9
	No evidence of disease	548	87.1
Final condition	Alive without the disease	561	89.2
	Alive with the disease	38	6.0
	Dead of the disease	12	1.9
	Dead of other causes	18	2.9

Tg, serum Thyroglobulin; TSH, serum Thyroid-Stimulant Hormone.

a 8 follicular carcinomas with Hürthle cells.

b At initial diagnosis; total number of patients submitted to the exam: 492.

c At initial diagnosis; total number of patients submitted to the exam: 622.

RAI was offered to 45.5% of Group 1 (188 patients) and to 94.4% of Group 2 (204 patients) patients after initial surgery. Among patients who developed structural disease, the proportions of additional RAI were 37% (10 patients) in Group 1 and 43.7% (21 patients) in Group 2.

In Group 1, 376 patients (91.0%) had no evidence of cervical lymph node nor distant metastases at the time of the first treatment. In this group, 27 (6.5%) presented structural relapses during the follow-up period. In Group 2, 131 (60.6%) patients had no evidence of cervical lymph node nor distant metastases at the time of first treatment. In this group, 49 (22.7%) individuals presented structural relapses during the follow-up period (p < 0.001 – Chi-Square test).

Patients with microscopic tumor invasion (Group 2) exhibited more aggressive clinicopathological features than those without local invasion (Group 1), including higher rates of follicular carcinoma (p < 0.001), tumors > 2 cm (p < 0.001), angiolymphatic invasion (p < 0.001), pN1 disease (p < 0.001), multicentricity (p < 0.001), elevated postoperative thyroglobulin > 10 ng/mL (p = 0.038), radioiodine-refractory disease (p = 0.002), structural recurrence (p < 0.001), distant metastases (p = 0.001), and one-year suppressed thyroglobulin >1 ng/mL (p < 0.001). Complete comparison data are shown in Table 2.

**Table 2:** Association between both groups and epidemiological, pathological, and clinical variables.

Variables	No Invasion (Group 1)		Minimal invasion (Group 2)		p-value (Chi-Square/ Fisher’s exact test)	OR (95% CI)
	n	%	n	%		
Age (year at diagnosis)						
<45	124	30.0	67	31.0	0.785^a^	
≥45 and <55	108	26.2	51	23.6		
≥55	181	43.8	98	45.4		
Sex						
Male	50	12.1	31	14.4	0.425^a^	1.22 (0.75–1.97)
Female	363	87.9	185	85.6		reference
Histology^a^						
Papillary	398	96.6	199	93.0	0.041^b^	reference
Follicular	14	3.4	15	7.0		2.14 (1.01–4.53)
Diameter (cm)						
≤2	334	80.9	136	63.0	<0.001^a^	reference
>2 and ≤4	57	13.8	51	23.6		1.82 (1.27–2.59)
>4	22	5.3	29	13.4		2.76 (1.54–4.93)
Angiolymphatic invasion	27	6.5	58	26.9	<0.001^a^	5.25 (3.21–8.59)
pN1	32	7.7	78	36.1	<0.001^a^	6.73(4.27–10.6)
M1	8	1.9	10	4.6	0.054^a^	2.46 (0.96–6.32)
Multicentricity	164	39.7	117	54.2	<0.001^a^	1.79 (1.29–2.50)
Postoperative Tg under TSH suppression						
<1 ng/mL	118	41.0	66	32.4	0.038^a^	reference
1‒10 ng/mL	130	45.1	94	46.1		2.86 (1.75–4.66)
>10 ng/mL	40	13.9	44	21.6		1.71 (1.06–2.73)
Iodine refractivity	10	5.2	29	14.3	0.002^a^	
Structural relapse	27	6.5	49	22.7	<0.001^a^	4.19 (2.54–6.94)
Distant relapse	14	3.4	21	9.7	0.001^a^	3.07(1.53–6.17)
Basal Tg 1-year after initial treatment (ng/mL)						
<0.2	318	77.9	151	70.6	<0.001^a^	
0.2 – 1	57	14.0	20	9.3		
1 – 10	25	6.1	24	11.2		
>10	8	2.0	19	8.9		
Cumulative structural disease-free survival at 112-months		92.4		77.7	<0.001^c^	

pN1, Initial pathological cervical lymph node metastasis; M1, Initial distant metastasis; Tg, Serum Thyroglobulin; OR, Odds Ratio.

a Papillary + Follicular carcinoma cases (3-patients) were excluded.

b Fischer exact test.

c Log-rank test.

In the univariate survival analyses, male sex, follicular thyroid cancer, tumors >2 cm, the presence of angiolymphatic invasion, lymph node metastasis or microscopic invasion, postoperative levels of serum thyroglobulin >1 ng/mL ‒ ATA biochemical incomplete response, as well as iodine refractory disease were variables associated with the risk of structural recurrence during the follow-up period. However, in the multivariate analysis (Cox regression), only male sex (HR = 1.961; 95% CI: 1.122–3.429; p = 0.018), postoperative levels of thyroglobulin >10 ng/mL (HR = 6.883; 95% CI: 2.734–17.324; p < 0.001), and radioiodine refractory disease (HR = 7.913; 95% CI: 4.530–13.824; p < 0.001) were independent variables and determinants of the risk of structural recurrence (Table 3).

**Table 3:** Survival and regression analyses considering time to structural relapse as outcome.

		Univariate analysis				Multivariate analysis		
Variables		Events/Total (number)	Cumulative Survival (60 months)	Cumulative Survival (120 months)	p-value (Log-rank test)	HR	95% CI	p-value (Cox regression)
Sex	Male	22 / 91	76.5%	68.9%	0.009	1.961	1.122‒3.429	0.018
	Female	84 / 591	87.2%	84.4%		Ref.		
Age (year at diagnosis)	<45	51 / 312	86.5%	79.8%	Ref.	Ref.		
	≥45 and <55	22 / 170	87.8%	87.8%	0.236	*	*	*
	≥55	33 / 200	84.8%	82.2%	0.923	*	*	*
Histology	Papillary	93 / 649	86.9%	83.5%	<0.001	Ref.		
	Follicular	13 / 30	59.6%	59.6%		1.324	0.561‒3.125	0.522
Diameter (cm)	<2	47 / 477	91.4%	91.4%	Ref.	Ref.		
	2‒4	27 / 130	80.5%	77.0%	<0.001	0.829	0.468‒1.468	0.520
	>4	32 / 75	59.7%	57.3%	<0.001	1.166	0.658‒2.066	0.599
Angiolymphatic invasion	No	52 / 562	91.4%	89.3%	<0.001	Ref.		
	Yes	53 / 119	59.9%	52.1%		1.575	0.971‒2.557	0.066
pN	0	22 / 224	91.8%	–	Ref.	Ref.		
	1a	28 / 312	91.4%	91.4%	<0.001	1.281	0.666‒2.463	0.458
	1b	26 / 78	68.6%	64.0%	<0.001	0.716	0.359‒1.430	0.344
Multicentricity	No	56 / 371	86.8%	82.6%	0.594	Ref.		
	Yes	50 /311	84.5%	82.7%		*	*	*
Local invasion	No	27 / 413	94.1%	92.4%	<0.001	Ref.		
	Minimal	46 / 213	79.2%	77.7%		0.987	0.541‒1.801	0.965
Postoperative Tg under TSH suppression (ng/mL)	<1	7 / 192	96.8%	95.7%	Ref.	Ref.		
	1‒10	18 /235	93.4%	88.3%	<0.001	2.000	0.782‒4.117	0.148
	>10	66 / 112	44.4%	‒	<0.001	6.883	2.734‒17.327	<0.001
Iodine refractivity	No	38 / 386	91.0%	88.2%	<0.001	Ref.		
	Yes	59 / 62	16.1%	6.3%		7.913	4.530‒13.824	<0.001

HR, Harzard Ratio; CI, Confidence Interval; Ref., Reference; pN, Pathological cervical lymph node metastasis; Tg, Serum Thyroglobulin; TSH, Serum Thyroid-Stimulant Hormone; Ref., Reference.

* Analysis not performed due to absence of significant association at univariate analyses.

At the end of the follow-up period, 377 (91.3%) patients from Group 1 were alive without structural disease, 16 (3.9%) were alive with evidence of structural disease (ATA structural incomplete response), 6 (1.4%) were dead from the disease, and 14 (3.4%) died from other causes. In Group 2, the proportions were 184 (85.2%), 22 (10.2%), 6 (2.8%), and 4 (1.8%), respectively, for those outcomes.

**Fig. 1:**
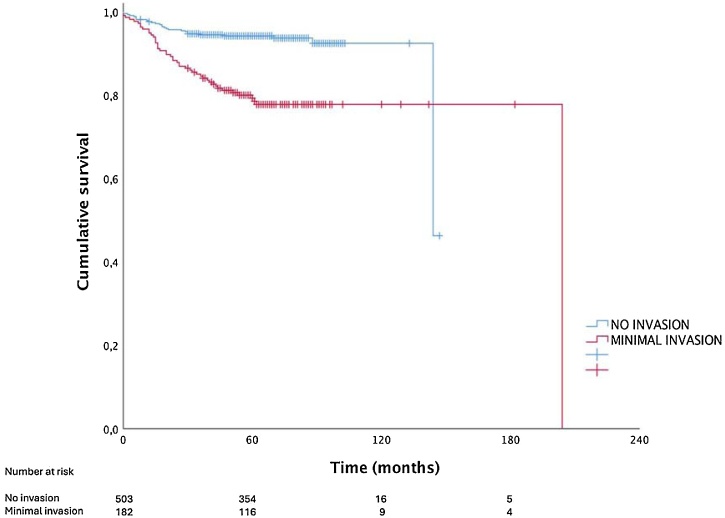
Structural disease-free survival curves for patients with differentiated thyroid carcinoma without extrathyroidal extension (Group 1) and with minimal invasion (Group 2). The Kaplan–Meier curves demonstrate evident curve discrimination between both groups (cumulative survival at 112-months: 92.4 vs. 77.7% for Groups 1 and 2, respectively; p < 0.001 – log-rank test).

Fig. 1 shows the Kaplan–Meier curves for SDFS for both groups. The cumulative survival rates were 92.4% and 77.7% for Groups 1 and 2, respectively, in at least 112-months of follow-up (p < 0.001; log-rank test).

To evaluate the potential impact of treatment differences on outcomes, a subgroup analysis was performed including only patients who received the same therapeutic approach (surgery followed by radioactive iodine therapy, n = 375). In this cohort, multivariate Cox regression analysis demonstrated that postoperative suppressed thyroglobulin >10 ng/mL (HR = 3.59; 95% CI: 2.045–6.32; p < 0.001) and radioiodine-refractory disease (HR = 11.276; 95% CI: 5.648–22.510; p < 0.001) remained independently associated with structural recurrence. In contrast, sex was no longer a significant factor in this subgroup (p = 0.206). These findings indicate that treatment differences did not substantially alter the primary associations identified. Full results of this subgroup analysis are provided in the Supplement.

## Discussion

The present findings indicate that minimal Extrathyroidal Extension (mETE) is a pathological feature frequently associated with other adverse prognostic variables in Differentiated Thyroid Carcinoma (DTC), but it may not be an independent predictor of poor outcome. Patients with mETE tumors exhibited a significantly higher rate of structural recurrence (22.7% vs. 6.5%, p < 0.001) and SDFS compared to non-invasive tumors (77.7% vs. 94.2% at 112-months; p < 0.001), although mETE was not independently associated with structural recurrence or SDFS in the multivariate analysis. These findings contribute to the ongoing discussion in current literature.

Although mETE was not independently associated with SDFS in the multivariate analysis, it showed a strong correlation with higher postoperative thyroglobulin levels. In our cohort, the prognostic impact of mETE appeared to be influenced by postoperative Tg: patients with mETE but low Tg had outcomes similar to those without mETE, whereas the combination of mETE and elevated Tg identified a subgroup at substantially higher risk of recurrence. These findings reinforce the importance of integrating both histopathological findings and biochemical response into risk stratification and follow-up decisions.

Historically, mETE upstaged DTC to pT3 in the 6th and 7th editions of the AJCC/UICC TNM staging system, and even to pT4 in the 5th edition. However, in the 8th edition (2017), mETE was excluded from TNM staging due to lack of independent prognostic significance.^9^

Nevertheless, the clinical relevance of mETE remains controversial. While some studies have found no association with increased mortality or recurrence risk,^14^^,^^17^, ^18^, ^19^, ^20^, ^21^, ^22^, ^23^, ^24^, ^25^ others have demonstrated that mETE correlates with a higher recurrence rate and impaired Disease-Free Survival (DFS), regardless of tumor size.^11^^,^^14^ A systematic review and meta-analysis further supported this view, showing that mETE is significantly associated with increased risk of disease recurrence.^16^

In this series, univariate analysis demonstrated that mETE was significantly associated with established risk factors such as angiolymphatic invasion, lymph node metastases (pN1), multicentricity, radioiodine refractoriness, and distant metastases. Patients with mETE also exhibited higher postoperative stimulated and basal thyroglobulin levels, indicating a greater tumor burden. These findings are consistent with previous studies reporting that mETE is linked to an increased likelihood of lymph node metastases, lymphovascular invasion, and larger tumor size.^13^ Additionally, patients with mETE experienced significantly higher recurrence rates compared to those without mETE. Other studies have further associated mETE with positive surgical margins, extranodal extension, lymphovascular invasion, and disease recurrence.^26^

However, mETE was not confirmed as an independent risk factor and several studies support this finding. In a matched-pair study of 160 patients (80-pairs), no significant difference in recurrence rates was found between patients with and without mETE, although those with mETE more frequently received external beam radiation and higher doses of radioiodine (p < 0.001), suggesting a potential treatment intensity bias.^27^ Other studies similarly reported no significant outcome differences,^28^^,^^29^ and several failed to identify mETE as an independent predictor of persistent disease, recurrence-free survival, or overall survival.^23^^,^^25^^,^^29^, ^30^, ^31^

Two retrospective studies also found no increased risk of death or recurrence associated with Mete.^5^^,^^17^ Tran et al.^11^ noted that mETE may function as a surrogate for other adverse features, such as lymphovascular invasion, multifocality, positive margins, pN1 status, and extranodal extension. In line with our findings, they reported that although mETE was associated with lower survival in univariate analysis, it was not an independent prognostic factor. The authors emphasized that including biological variables as prognostic markers solely based on their association with other risk factors is inconsistent with principles of evidence-based medicine.

Extensions to the glandular surgical capsule, perithyroidal soft tissue, or sternothyroid muscle are classified as minimal Extrathyroidal Extension (mETE), occurring in 15%–66% of Differentiated Thyroid Carcinomas (DTC) measuring 1–4 cm.^13^ This wide range likely reflects the lack of a standardized definition for minimal invasion. Diagnosis of mETE can be subjective, as it is not a well-defined histopathologic feature, and the thyroid gland lacks a true anatomic capsule.^17^

Therefore, histopathological interpretation is subject to interobserver variability, affecting both the reported rates of mETE and its prognostic implications, contributing to difficulty in establishing its prognostic significance. In a study involving 11 expert endocrine pathologists, there was no consensus on features such as tumor extension into perithyroidal fat, nerves, thick-walled vessels, or skeletal muscle.^32^

The 2015 ATA Guidelines classified mETE as an intermediate-risk feature for recurrence, potentially influencing decisions regarding adjuvant Radioiodine (RAI) therapy.^10^^,^^18^ In our cohort, adjuvant RAI therapy was administered to 45.5% of patients without mETE and 94.4% of those with mETE, reflecting temporal changes in guidelines and institutional practice. Differences in recurrence rates between patients who did and did not receive RAI should be interpreted with caution, as they likely reflect treatment selection and not necessarily causal effects of RAI.

A reasonable clinical approach to guide the indication of Radioiodine therapy (RAI) in patients presenting mETE as an isolated risk factor may involve individualized risk stratification after surgery. This should include the assessment of initial response to therapy ‒ such as serum thyroglobulin levels and neck ultrasound findings ‒ rather than relying solely on the presence of Mete.^18^^,^^33^ Ongoing follow-up and treatment decisions should consider mETE within the broader context of tumor biology and other coexisting prognostic factors.

This study has several limitations inherent to its retrospective, single-center design, including potential selection and information biases. The definition of mETE may have varied among pathologists over the study period due to the absence of a centralized pathology review, and treatment strategies, particularly the indication for RAI, were not uniform, especially given the long inclusion period. This may have introduced time effects, as treatment patterns and follow-up strategies evolved substantially during this interval. These limitations are intrinsic to retrospective studies, in which it is difficult to obtain standardized and robust data across long observation periods; nevertheless, they reflect real-world clinical practice.

In conclusion, mETE was significantly associated with structural recurrence in univariate analysis but was not an independent prognostic factor in multivariate models, where postoperative Tg emerged as the strongest predictor of outcome. These findings highlight the importance of integrating histopathological features and biochemical response in risk stratification, but further studies are needed to clarify their implications.

## ORCID ID

Beatriz G Cavalheiro: 0000-0001-9720-5034

Vergilius José Furtado de Araújo Filho: 0000-0001-7027-5681

Leandro Luongo Matos: 0000-0002-5068-8208

Ana Kober Leite: 0000-0001-7814-757X

Claudio Roberto Cernea: 0000-0001-5899-0535

Luiz Paulo Kowalski: 0000-0002-0481-156X

## Declaration of Generative AI and AI-assisted technologies in the writing process

During the preparation of this work the author(s) used ChatGPT (OpenAI) for general writing revision and language refinement. After using this tool, the authors reviewed and edited the content as needed and take full responsibility for the content of the publication.

## Funding

This research received no external funding.

## Data availability statement

The authors declare that all data are available in repository.

## Declaration of competing interest

The authors declare no conflicts of interest.
