# Supplementary material for: Prognostic significance of minimal extrathyroidal extension in differentiated thyroid carcinoma: a retrospective cohort study
Source: Braz J Otorhinolaryngol. 2026 Mar 11;92(3):101780. doi: 10.1016/j.bjorl.2026.101780 (PMC12996654; doi:10.1016/j.bjorl.2026.101780)
Supplement: Supplementary file 1 [file mmc1.docx]

BJORL-D-25-00141

**Supplement**

**Table 1** Survival and regression analyses considering time to structural relapse as outcome among patients treated with surgery and radioiodine therapy.

|  | | **Univariate analysis** | | **Multivariate analysis** | | |
| --- | --- | --- | --- | --- | --- | --- |
| **Variables** | | **Events / Total (number)** | **p-value (Log-rank test)** | **HR** | **95% CI** | **p-value (Cox regression)** |
| Sex | Male | 11 / 48 | 0.206 |  |  |  |
|  | Female | 55 / 341 |  |  |  |  |
| Age (year at diagnosis) | < 45 | 25/124 | Ref. |  |  |  |
|  | ≥ 45 and < 55 | 13/91 | 0.656 |  |  |  |
|  | ≥ 55 | 28/174 | 0.356 |  |  |  |
| Histology | Papillary | 56/362 | <0.001 | Ref. |  |  |
|  | Follicular | 10/25 |  | 0.857 | 0.321‒2.286 | 0.758 |
| Diameter (cm) | < 2 | 38/240 | Ref. | Ref. |  |  |
|  | 2‒4 | 13/101 | 0.52 | 0.737 | 0.355‒1.533 | 0.415 |
|  | > 4 | 15/48 | 0.005 | 0.836 | 0.412‒1.695 | 0.619 |
| Angiolymphatic invasion | No | 37/306 | <0.001 | Ref. |  |  |
|  | Yes | 29/83 |  | 1.299 | 0.741‒2.278 | 0.361 |
| pN | 0 | 34 / 282 | Ref. | Ref. |  |  |
|  | 1a | 18 / 62 | 0.003 | 1.6 | 0.768‒3.339 | 0.210 |
|  | 1b | 14 / 45 | 0.006 | 0.61 | 0.268‒1.391 | 0.240 |
| Multicentricity | No | 36/181 | 0.212 |  |  |  |
|  | Yes | 30/208 |  |  |  |  |
| Local invasion | No | 21 / 188 | 0.006 | Ref. |  |  |
|  | Minimal | 45 / 201 |  | 0.749 | 0.393‒1.427 | 0.379 |
| Postoperative Tg under TSH suppression (ng/mL) | < 1 | 3/128 | Ref. | Ref. |  |  |
|  | 1‒10 | 13/174 | 0.045 |  |  |  |
|  | > 10 | 41 / 75 | <0.001 | 3.59 | 2.045‒6.32 | <0.001 |
| Iodine refractivity | No | 28 / 348 | <0.001 | Ref. |  |  |
|  | Yes | 37 / 39 |  | 11.276 | 5.648‒22.510 | <0.001 |

HR, Harzard Ratio; CI, Confidence Interval; Ref., Reference; pN, Pathological Cervical lymph node metastasis; Tg, Serum Thyroglobulin; TSH, Serum Thyroid-Stimulant Hormone.

Analysis not performed due to absence of significant association at univariate analyses.
